# Supplementary material for: Cell Count Differentials by Cytomorphology and Next-Generation Flow Cytometry in Bone Marrow Aspirate: An Evidence-Based Approach
Source: Diagnostics (Basel). 2023 Mar 11;13(6):1071. doi: 10.3390/diagnostics13061071 (PMC10047335; doi:10.3390/diagnostics13061071)
Supplement: Supplementary file 1 [file diagnostics-13-01071-s001.zip › diagnostics-2242335-supplementary.pdf]

CELL COUNT DIFFERENTIALS BY CYTOMORPHOLOGY AND NEXT-GENERATION FLOW CYTOMETRY IN BONE MARROW ASPIRATE: AN EVIDENCE-BASED APPROACH

SUPPLEMENTARY MATERIAL

Table S1. Monoclonal antibodies reagents used for the immunophenotypic characterization.

Mab	Conjugates
CD2	FITC
HLA-DR	FITC
CD11b	FITC
CD14	PE
CD34	PE
CD13PE	PE
CD4	PerCP-Cy5.5
CD10	PerCP-Cy5.5
HLA-DR	PerCP-Cy5.5
CD7	APC
CD33	APC
CD34	APC
CD56	PE-CY7
CD19	PE-CY7
CD117	PE-CY7
CD8	APC-H7
CD38	APC-H7
CD71	APC-H7
CD3	V450
CD20	V450

CD15	V450
CD45	V500

Abbreviations: AF: alexa fluor; APC: allophycocyanin; BB: brilliant blue 515; BV: brilliant violet, FITC: fluorescein isothiocyanate; Mab: monocclonal antibodies; PE: phycoerythrin; PerCP-Cy5.5: peridinin chlorophyll protein-cyanine 5.5.

Table S2. Final diagnosis in the complete set and the control group, %.

DISEASE	COMPLETE SET n=660	CONTROL GROUP n=122
AML	17.6	25.4
MM	15.6	24.6*
MDS	15.5	4.1
LYM	14.2	16.4
MGUS	10.5	*
ALL	7.6	5.7
CMPD	4.5	7.4
OTHER	14.5	16.4

Abbreviations: ALL: acute myeloid leukemia; AML: acute myeloid leukemia; CMPD: chronic myeloproliferative disease; LYM: lymphomas; MGUS: monoclonal gammopathy of uncertain significance; MM: multiple myeloma. *this percentage is presented together with MM in the control group.

Table S3. Comparison by Regression Linear analysis between BMCS 200-cell and 500-cell DCC versus IP FDC results for the complete set according to cell type*

CELL TYPE	n	R^2	Slope	Intercept
Blast 200	424	0.863	0,939	-,051
Blast 500	424	0.897	0,960	-,033
Promyelocyte 200	102	0.955	1,007	-,763
Promyelocyte 500	102	0.966	1,006	-,675
Erythroblast 200	189	0.453	1,221	12,401
Erythroblast 500	189	0.465	1,201	13,774
Lymphocyte 200	235	0.590	0,628	,895
Lymphocyte 500	235	0.650	0,726	-,093
Plasma cell 200	343	0.921	1,436	-,026
Plasma cell 500	343	0.917	1,427	-,002
Monocyte 200	390	0.662	1,081	-,063
Monocyte 500	390	0.683	1,115	-,471

*All associates p values are significant at <.000

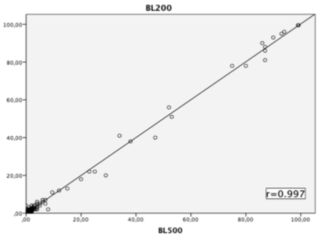

Ecuación	Resumen del modelo					Estimaciones de los parámetros	
	R cuadrado	F	gl1	gl2	Sig.	Constante	b1
Lineal	,995	102277,124	1	549	,000	-,104	1,003

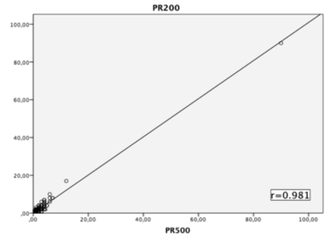

Ecuación	Resumen del modelo					Estimaciones de los parámetros	
	R cuadrado	F	gl1	gl2	Sig.	Constante	b1
Lineal	,962	14021,366	1	549	,000	-,013	1,009

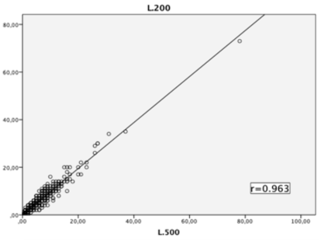

Ecuación	Resumen del modelo					Estimaciones de los parámetros	
	R cuadrado	F	gl1	gl2	Sig.	Constante	b1
Lineal	,926	6899,826	1	548	,000	,124	,966

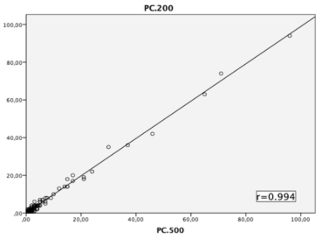

Ecuación	Resumen del modelo					Estimaciones de los parámetros	
	R cuadrado	F	gl1	gl2	Sig.	Constante	b1
Lineal	,989	48657,100	1	548	,000	-,038	,989

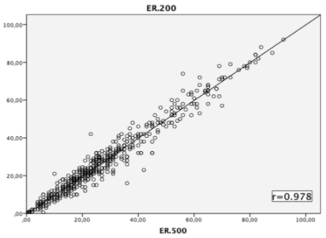

Ecuación	Resumen del modelo					Estimaciones de los parámetros	
	R cuadrado	F	gl1	gl2	Sig.	Constante	b1
Lineal	,957	12055,095	1	548	,000	-,283	1,001

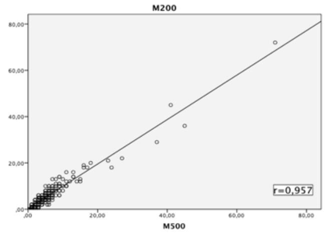

Ecuación	Resumen del modelo					Estimaciones de los parámetros	
	R cuadrado	F	gl1	gl2	Sig.	Constante	b1
Lineal	,916	5945,487	1	548	,000	,330	,960

Figure S1. Linear Regression analysis for the complete set, 200-cell vs 500-cell DCC

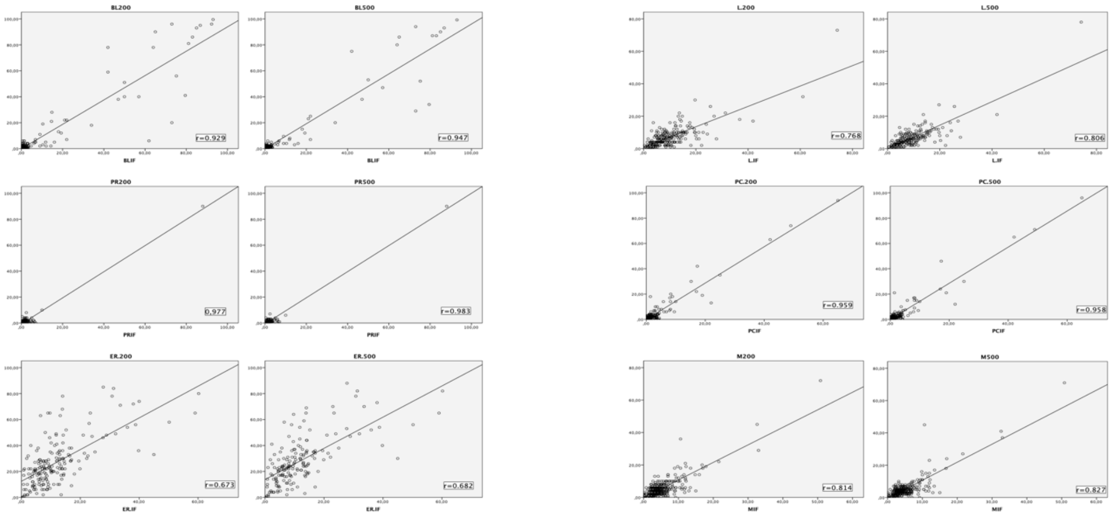

Figure S2. Linear Regression analysis for the complete set comparison between 200-cell and 500-cell DCC and immunophenotype
